# Randomized Controlled Trial of Automated Directly Observed Therapy for Measurement and Support of PrEP Adherence Among Young Men Who have Sex with Men

**DOI:** 10.1007/s10461-022-03805-3

**Published:** 2022-08-19

**Authors:** Susan P. Buchbinder, Aaron J. Siegler, Kenneth Coleman, Eric Vittinghoff, Gretchen Wilde, Annie Lockard, Hyman Scott, Peter L. Anderson, Nicole Laborde, Ariane van der Straten, Richard H. Christie, Michelle Marlborough, Albert Y. Liu

**Affiliations:** 1 Bridge HIV, San Francisco Department of Public Health, San Francisco, CA USA; 2 Department of Medicine, University of California San Francisco, San Francisco, CA USA; 3 Department of Epidemiology and Biostatistics, University of California, San Francisco, CA USA; 4 Department of Epidemiology, Emory University, Atlanta, GA USA; 5 University of Colorado Anschutz Medical Campus, Aurora, CO USA; 6 Independent Consultant, San Rafael, USA; 7 ASTRA Consulting, Kensington, CA USA; 8 AiCure, New York, NY USA; 9 Bridge HIV, San Francisco Department of Public Health, 25 Van Ness Avenue, Suite 100, San Francisco, CA 94102 USA

**Keywords:** Men who have sex with men (MSM), Pre-exposure prophylaxis (PrEP), Adherence, Directly observed therapy (DOT), Artificial intelligence (AI)

## Abstract

Measurement of adherence to oral pre-exposure prophylaxis (PrEP) in real-time has been challenging. We developed DOT Diary, a smartphone application that combines automated directly observed therapy with a PrEP adherence visualization toolkit, and tested its ability to measure PrEP adherence and to increase adherence among a diverse cohort of young men who have sex with men (MSM). We enrolled 100 MSM in San Francisco and Atlanta and randomly assigned them 2:1 to DOT Diary versus standard of care. Concordance between DOT Diary measurement and drug levels in dried blood spots was substantial, with 91.0% and 85.3% concordance between DOT Diary and emtricitabine-triphosphate and tenofovir-diphosphate, respectively. There was no significant difference in the proportion of participants with detectable PrEP drug levels at 24 weeks between study arms. These results suggest DOT Diary is substantially better than self-reported measures of adherence, but additional interventions are needed to improve PrEP adherence over time.

## Resumen

La medición de la adherencia a la profilaxis oral previa a la exposición (PrEP) en tiempo real ha constituido un desafío. Hemos desarrollado DOT Diary, una aplicación para teléfonos inteligentes que combina la terapia automatizada observada de forma directa con un kit de herramientas para visualizar la adherencia a la PrEP, y testeamos su capacidad para medir la adherencia a la PrEP, así como para aumentar la adherencia entre una cohorte variada de hombres jóvenes que tienen sexo con hombres (HSH). Reclutamos a 100 HSH en San Francisco y Atlanta y los asignamos aleatoriamente 2:1 a DOT Diary con respecto a la asistencia estándar. La concordancia entre la medición del DOT Diary y los niveles de fármacos en gotas de sangre seca fue sustancial, con un 91,0% y un 85,3% de concordancia entre el uso del DOT Diary y el de emtricitabina-trifosfato y tenofovir-difosfato, respectivamente. No hubo diferencias significativas en la proporción de participantes con niveles detectables del fármaco de la PrEP a las 24 semanas entre los brazos del estudio. Estos resultados sugieren que DOT Diary es sustancialmente superior a las medidas de adherencia que se notifican de forma personal, aunque hacen falta intervenciones adicionales para mejorar la adherencia a la PrEP a largo plazo.

## Introduction

Men who have sex with men (MSM) remain the population with the largest number of HIV infections in the United States, accounting for 70% of new HIV diagnoses in 2019 [1]. Young MSM, aged 25–34 years account for the largest number of new diagnoses compared with other age groups. Black/African American MSM accounted for more than half of all new diagnoses among 13–24 year old MSM, and for one third of all new diagnoses among MSM > 24 years old. Latino MSM are also disproportionately affected by HIV, accounting for 33% of all new diagnoses among MSM. Clearly, highly effective prevention strategies are needed for young Black/African American and Latino MSM.

Oral pre-exposure prophylaxis (PrEP) with tenofovir disoproxil fumarate co-formulated with emtricitabine (TDF/FTC) has been shown to be highly effective in MSM in several randomized controlled trials [2, 3], demonstration projects [4, 5], and in clinical practice [6]. However, PrEP persistence (continuing treatment over time) has been challenging, with 37–62% of MSM discontinuing PrEP use by 6 months [7–9], and young and Black/African-American and Latinx MSM more likely to quit [10–12]. Measurement of adherence has also been challenging, with substantial over-reporting of PrEP use by self-report [13, 14]. Real-time objective measures are needed to measure adherence patterns during clinical trials, and to assist clinicians to counsel patients appropriately about their PrEP adherence. To date, the most common real-time adherence method used in PrEP trials has been electronic medication containers such as MEMSCap and WisePill [15, 16]. However, these devices have been found to correlate poorly with biologic measures [16–19]. This is likely because they do not directly confirm pill ingestion, can be defeated easily by extra openings without ingestion, and data can be lost by faulty transmission or loss of devices. Software-based methods that leverage existing smartphones and do not require changes to the medication itself [20] can facilitate real-time monitoring and feedback.

Because of its ability to ensure treatment adherence, directly observed therapy (DOT) has been used for decades both to measure and maximize adherence for treatment of tuberculosis infection, and more recently to increase adherence to antiretrovirals for treatment [21], and to ensure protocol-defined dosing in PrEP pharmacokinetics (PK) studies [22, 23]. Several studies have found DOT to be successful in improving adherence to antiretroviral therapy [24, 25], including in Black/African American and Latinx populations [26, 27]. However, the cost and logistical complexity of DOT in large clinical trials can be prohibitive and in clinical practice, it is impractical. Automated DOT (a-DOT), using artificial intelligence with advanced features to detect intentional non-adherence, combines the accuracy of in-person DOT with the convenience of real-time centralized data collection and monitoring. Addition of a PrEP adherence visualization toolkit, including a daily sexual diary, expands the applicability of this tool for assessing intermittent PrEP regimens.

We developed DOT-Diary, a smartphone application (app) that combines a-DOT with a PrEP adherence visualization toolkit, and conducted a randomized clinical trial to assess its ability to accurately measure PrEP adherence and to increase adherence among a racially/ethnically diverse cohort of young MSM.

## Methods

### Study Population

To be eligible to enroll, participants had to self-identify as a man aged 18–35, and report having insertive or receptive anal sex with a man or transgender woman in the prior 12 months. Men also had to report any one of the following in the prior 12 months: (1) any condomless anal sex outside of a mutually monogamous relationship with an HIV-negative partner; (2) two or more anal sex partners; (3) self-reported gonorrhea, chlamydia or syphilis; or (4) having a known HIV-positive sex partner. Men also had to be HIV-negative as determined by a negative 4th generation HIV test, be willing and eligible to take TDF/FTC PrEP, have a creatinine clearance of ≥ 60 ml/min and be hepatitis B surface antigen negative. Because the DOT Diary app was only available in English, participants had to be able to read and speak English, and to own a smartphone that was compatible with the DOT Diary application (iOS or Android). Participants were excluded for any of the following reasons: (1) PrEP use in the previous 4 months; (2) any reactive HIV test or signs or symptoms of acute HIV at screening or enrollment; (3) history of pathologic bone fracture; or (4) taking nephrotoxic medications. Participants were drawn from the Atlanta Metropolitan Area or the greater San Francisco Bay Area. We enrolled 100 participants from February to May 2019, evenly divided between the Atlanta and San Francisco sites.

All phases of this study were approved by the University of California San Francisco Institutional Review Board, which operated as a single IRB under a reliance agreement with Emory University, and this trial was registered on clinicaltrials.gov [NCT03771638].

### Intervention Components

Participants were randomized 2:1 to DOT Diary versus a standard of care arm. Participants in both arms of the trial received daily oral TDF/FTC dispensed in 30-pill bottles at enrollment and week 12. Participants were instructed to take one pill orally once daily, and extra bottles were dispensed to ensure the participant had sufficient drug in case of delays before their next visit. Visits were scheduled every 6 weeks through 24 weeks of follow-up. All participants also received risk reduction counseling and access to condoms and lubricant at each visit. Participants answered questions by computer-assisted self-interview (CASI) about their demographics (baseline), their sexual practices, past history of sexually transmitted infections, and drug and alcohol use (baseline and at weeks 12 and 24), and the positive and negative impacts of study participation (week 24). Participants randomized to the DOT Diary arm also answered questions about diary use and acceptability at weeks 12 and 24. The System Usability Scale (SUS) [28] includes ten questions regarding app usability, with a 5-point Likert scale from strongly agree to strongly disagree; answers are presented combining the strongly agree and agree categories and the strongly disagree and disagree categories. For the SUS, a score of > 80.3 is considered an “A” grade score, and a score of 68.0–80.3 is considered a “B” grade score. The Client Satisfaction Survey (CSQ-8) [29] consists of eight questions to assess client/patient satisfaction with services provided, using a 4-point Likert scale; it was adapted for use with the DOT Diary app. For the CSQ-8, a score of < 20 is considered “low,” 21–26 is considered “medium,” and 27–32 is considered “high.”

Participants received 4th generation HIV testing and STI testing (pharyngeal and rectal swabs and urine collection for gonorrhea and chlamydia testing, and blood for syphilis testing) at screening, week 12, and week 24 of the study. Dried blood spots (DBS) to test for tenofovir diphosphate (TFV-DP) and emtricitabine triphosphate (FTC-TP) concentrations were collected at weeks 6, 12, 18, and 24.

Participants randomized to the intervention arm received the DOT Diary application, previously described [30]. In collaboration with AiCure (New York, NY), we created DOT Diary for monitoring and supporting PrEP use. Briefly, DOT Diary is downloaded onto the participant’s smartphone and is Health Insurance Portability and Accountability Act (HIPAA)-compliant. Participants show their pill to their smartphone camera, place the pill on their tongue, and then swallow the pill. Computer vision and neural networks confirm that the correct participant is presenting the correct medication and ingesting the correct medication. The computer vision and algorithms have been optimized over time through machine learning and assessment of the visual and audio data collected from participants dosing on the platform on an ongoing basis. Real-time data (dosing data and patient reported outcomes) are transmitted to a centralized, cloud-based system for analysis and monitoring.

Participants received daily dosing reminder alarms, at the time of their choosing, prompting them to go into the app and visually confirm taking their medication. Participants received automated messages if they were late in taking a given dose, and study staff could also select from a list of pre-approved text messages to send to participants to follow-up on missed or late doses. In addition to a date and time stamp of each dosing event, doses were automatically classified as taken or missed; if they forgot or were unable to use the system to confirm their dosing, participants could self-report doses through a button within the application. Doses indicated by self-report are included in the total dosing data and classified as ingested doses. The artificial intelligence system flagged certain doses for further review if it detected signs of intentional nonadherence, such as presenting a different pill, spitting out the medication, or hiding it in the mouth without swallowing; if it was confirmed upon review that the medication ingestion did not occur, study staff were notified with a “red alert.” Study staff contacted participants for red alerts to determine the cause for the red alerts and try to mitigate any further problems with app use.

**Fig. 1:**
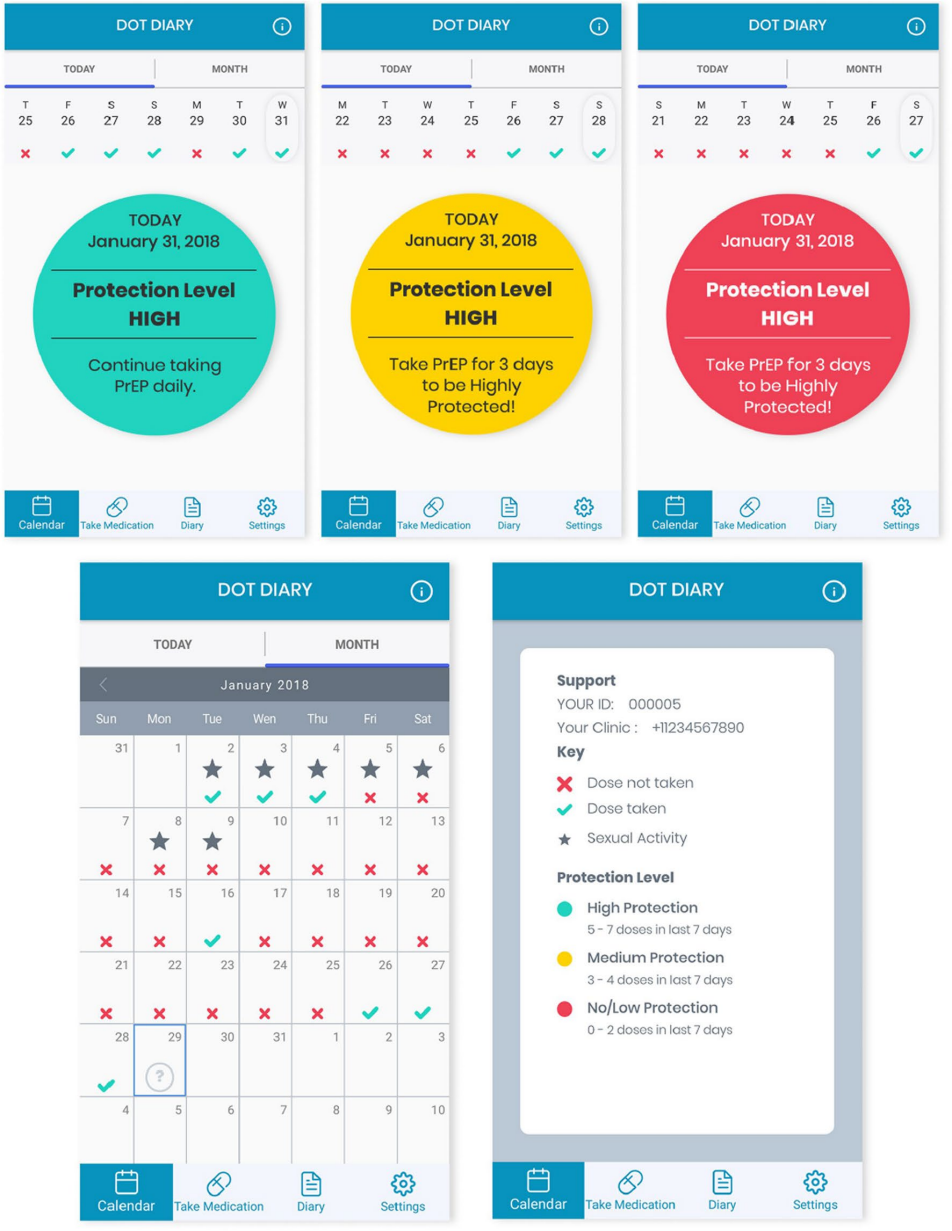
Features of the DOT Diary app

To facilitate participant tracking of sexual encounters, a sexual diary was integrated into the app. The sexual diary allowed participants to track sexual behaviors that occurred in each sexual encounter as well as an optional rating of partner characteristics, for the participants use; the latter was encrypted and not transmitted to the study team to protect privacy. The study app provided a calendar displaying all days in which PrEP medication was taken, and all days in which sexual activity occurred, allowing participants to see coverage of sexual encounters with PrEP (Fig. 1). Based on PK and pharmacodynamic (PD) data from prior PrEP trials [22, 31, 32], the app also indicated the estimated level of protection achieved from PrEP (i.e., low, medium, high) on the home screen, along with a record of their pill-taking in the past week, and motivating personalized messages on the number of additional PrEP doses needed to maximize or maintain maximal protection. Participants received a micro-incentive ($0.50) each day DOT Diary was used for dosing. To evaluate the DOT Diary app independent of the use of micro-incentives, participants randomized to the control condition also received $0.50 per day for each day they used a health-related app of their choosing on their phone (e.g., step count, sleep tracking).

At the end of the study, all participants were linked to PrEP services. For participants experiencing a delay in start-up of PrEP, an additional 3-month supply of PrEP was provided for bridging, to ensure no participant had a lapse in receiving PrEP at study end.

### Laboratory Measurement

A 3 mm punch was extracted from each DBS, and TFV-DP and FTC-TP were measured, using validated liquid chromatography-tandem mass spectrometry, as previously described [32, 33].

### Statistical Analyses

Baseline characteristics of control and intervention participants were summarized using descriptive statistics and compared using *t-*, Wilcoxon, χ^2^ and Fisher’s exact test as appropriate. GEE Poisson models with robust standard errors were used to compare marginal study retention and DBS adherence rates, averaged across visits. Concordance between TFV-DP and FTC-TP in DBS with adherence as measured by DOT Diary was calculated as the percentage of observations that were concordant (both positive or both negative) and corrected for chance agreement using the kappa statistic. Because of the differential half-lives of these two metabolites in DBS, we evaluated at least four times per week dosing of TDF/FTC as measured by DOT Diary (automated, by phone, or by self-report) in the 6 weeks prior to each TFV-DP DBS measurement (using a ≥ 700 fmol/punch cutoff), and any dosing of TDF/FTC as measured by DOT Diary in the 2 days prior to having any detectable FTC-TP in DBS [22]. The 700 fmol/punch cutoff was used as a marker of adherence associated with high efficacy in MSM [34]. Acceptability of DOT Diary, ratings of various components of the app, and PrEP coverage of self-reported sexual episodes were summarized using appropriate descriptive statistics. GEE logistic models were used to assess independent correlates of protective levels, identified via an initial screening step, followed by backward selection.

## Results

One hundred participants were enrolled in the study; 34 were randomized to the control condition, and 66 to the intervention arm, with most baseline demographic and risk variables comparable between the two groups (Table 1). Mean age was 26 years, with substantial racial/ethnic diversity. The majority of participants were college graduates and engaged in full-time work, although more than half reported an annual income below $50,000. Three-quarters of the participants had health insurance; being privately insured was most common. Binge drinking and marijuana use in the 6 months prior to enrollment were common. More than 1/3 of all participants reported popper use, but cocaine/crack and methamphetamine use were uncommon. More than 2/3 of participants reported receptive anal sex in the 3 months prior to enrollment; approximately half reported not using condoms. Approximately 1/4 of participants reported having had a bacterial STI in the 3 months prior to enrollment. Despite these risk characteristics, prior PrEP use was uncommon in this cohort. Overall, 74% of participants reported previously using an app to track health behaviors and 70% reported using an app for medication reminders, with no significant difference between the two study arms.

**Table 1:** Baseline demographic characteristics and HIV risk behavior in previous 3 months of enrolled participants, by study arm

Characteristic	Control N = 34	Intervention N = 66	χ^2^	P value
Race/Ethnicity			1.982	0.74
Asian/Pacific-Islander	11.8%	15.2%		
Black/African-American	20.6%	28.8%		
Latinx	20.6%	22.7%		
White	44.1%	31.8%		
Other	2.9%	1.5%		
Sexual orientation			2.251	0.32
Bisexual	17.6%	7.8%		
Gay	79.4%	87.5%		
Queer	2.9%	4.7%		
Highest level of education			4.472	0.21
High school/GED	8.8%	10.6%		
Some college	23.5%	42.4%		
College graduate	52.9%	33.3%		
Post-graduate studies	14.7%	13.6%		
Employment status			5.733	0.22
Full-time work	67.6%	50.0%		
Part-time work	20.6%	19.7%		
Student	8.8%	25.8%		
Unemployed	2.9%	1.5%		
Other	0%	3.0%		
Annual pre-tax income			3.858	0.43
< $20,000	26.5%	28.1%		
$20,000–$49,999	32.4%	35.9%		
$50,000–$74,999	23.5%	12.5%		
$75,000+	5.9%	15.6%		
Don’t know/declines to answer	11.8%	7.8%		
Has health insurance	73.5%	78.1%	1.315	0.73
Health insurance type^a^			0.544	0.91
Private/HMO	44.1%	48.4%		
Government	17.6%	15.6%		
Other	11.8%	7.8%		
Don’t know	26.5%	28.1%		
Binge alcohol (≥ 6 drinks on one occasion)			1.197	0.75
Never	32.4%	39.4%		
< Monthly	35.3%	25.8%		
Monthly	20.6%	19.7%		
Daily or weekly	11.8%	15.2%		
Marijuana use	47.1%	66.7%	3.595	0.06
Popper use	26.5%	47.0%	3.929	0.047
Cocaine/crack use	14.7%	12.3%	0.113	0.74
Meth/speed/amphetamine use	2.9%	4.6%	0.161	0.69
Mean # male/transgender anal sex partners	2.8	4.5	t = − 1.723	0.09
Any receptive anal sex	70.6%	66.7%	0.159	0.69
Any receptive anal sex without condoms	52.9%	45.5%	0.504	0.48
Any insertive anal sex	88.2%	63.6%	6.735	0.009
Any insertive anal sex without condoms	44.1%	40.9%	.095	0.76
Diagnosed with gonorrhea, chlamydia, syphilis	29.4%	22.7%	0.535	0.46
Ever on PrEP	14.7%	14.3%	0.003	0.96

^a^Among those with health insurance

Retention in the study was high, with 94% and 91% of participants in the intervention and control conditions attending the 6-week visit, 83% and 91% attending the 12-week visit, 86% and 85% attending the 18-week visit, and 91% and 85% attending the 24-week visit respectively (relative risk 1.00).

### Primary Outcome: Concordance of DBS and DOT Diary Adherence Measures

As shown in Tables 2 and 3, the observed concordance between DOT Diary measurement of PrEP use and FTC-TP measured in DBS was 91.0%. Of 19 discordant observations, 12 (63%) were positive by DOT Diary but negative by DBS. The kappa for FTC-TP levels was 0.66, indicating substantial agreement in this measure [35]. Concordance of DOT Diary measurement of PrEP use and TFV-DP protective levels was also high, with an observed concordance of 85.3% and a kappa of 0.61, indicating substantial agreement. Of 31 discordant observations, 20 (65%) were positive by DOT but negative by DBS. We repeated the analyses limiting the measurement of dosing to only episodes in which the AI registered correct ingestion of the medication without a substantial difference in the outcomes.

**Table 2:** Concordance of FTC-TP in DBS with PrEP use in past 2 days as measured by DOT Diary

	DOT Diary No PrEP taken	DOT Diary Any PrEP taken	Total
FTC-TP below limit of quantification	24	7	31
FTC-TP detected	12	168	180
Total	36	175	211

Agreement: observed 91.0% expected 73.3% Kappa 0.66 (95% CI 0.53–0.80)

**Table 3:** Concordance of TFV-DP in DBS with PrEP use in prior 6 weeks as measured by DOT Diary

	DOT Diary PrEP use < 4 days/week	DOT Diary PrEP use 4–7 times/week	Total
TFV-DP < 700 fmol/punch	37	20	57
TFV-DP ≥ 700 fmol/punch	11	143	154
Total	48	163	211

Agreement: observed 85.3% expected 62.5% Kappa 0.61 (95% CI 0.47–0.74)

### Primary Outcome: Effect on PrEP Adherence

**Fig. 2:**
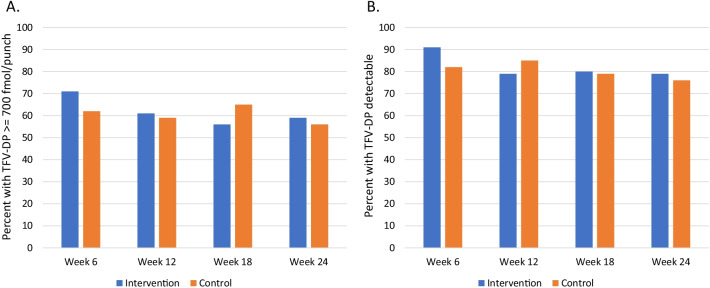
**A** Percent of participants with TFV-DP ≥ 700 fmol/punch; those missing DBS were counted as < 700 fmol/punch. **B** Percent of participants with detectable TFV-DP; those missing DBS were counted as undetectable

There was no significant difference in the proportion of participants with protective TFV-DP levels (≥ 700 fmol/punch) in DBS at the 6-, 12-, 18-, and 24-week visits by study arm (Fig. 2A), nor was having any TFV-DP detected in DBS different between groups (Fig. 2B). Only a median of 1 dose per participant was taken by self-report, using the app. Adjusted GEE Poisson models were evaluated for protective levels of TFV-DP, comparing intervention and control participants, with participants with missing DBS measures counted as < 700 fmol/punch; no difference was seen (RR 1.02, 95% CI 0.77–1.36). Similarly, the adjusted GEE Poisson model for detectable TFV-DP levels was also not significant (RR 1.02, 95% CI 0.86–1.20). These results were not different when restricting the analyses to only retained participants. We performed subgroup analyses with stratified models by site of recruitment, participant age ≤ 25, race/ethnicity, and prior PrEP use; none of these subgroup analyses yielded significant results. In a multivariable GEE model that evaluated demographic and risk variables associated with having protective levels of TFV-DP (≥ 700 fmol/punch), being enrolled in Atlanta (aOR 0.36, 95% CI 0.14–0.90) and Black/African American participants compared with White participants (aOR 0.25, 95% CI 0.09–0.72) were significantly less likely to have protective levels; persons with any receptive anal sex without condoms were significantly more likely to have protective levels (aOR 2.95, 95% CI 1.34–6.50). There were no HIV seroconversions during the study, but 8.1% were diagnosed with gonorrhea, chlamydia, or syphilis at the 12-week visit and 10.1% at the 24-week visit (overall STI incidence 16.8/100 person-years, 95% CI 9.6–27.2).

### Primary Outcome: Acceptability and Ease of Use of DOT Diary

Among participants randomized to the intervention arm, acceptability and ease of use of DOT Diary did not differ substantially at the week 12 and 24 visits (Table 4). Of the 60 intervention arm participants who were seen at the week 24 visit, the median SUS summary score was 80 (interquartile range (IQR) 72.5–87.5), which is at the cusp between an “A” and “B” grade score. The CSQ-8 total score was a median of 26.5 (IQR 24–31) at week 24, at the cusp between a “medium” and “high” score. In this questionnaire, participants were asked about their overall satisfaction with the app; 91.7% indicated that they were mostly or very satisfied with it. When asked if DOT Diary helped them in taking PrEP, 46.7% said it helped, and another 46.7% said it helped a great deal. When asked if they would use DOT Diary in the future, 96.6% responded that they would, and 90.0% said they would recommend the app to a friend.

**Table 4:** SUS responses among participants in the intervention arm

Question	12 weeks (n = 55) %	24 weeks (n = 60) %
Would like to use DOT Diary regularly		
Agree	79.6	75.0
Neutral	7.4	15.0
Disagree	13.0	10.0
DOT Diary is too complex		
Agree	7.4	8.3
Neutral	16.7	10.0
Disagree	75.9	81.7
DOT Diary is easy to use		
Agree	87.0	85.0
Neutral	7.4	10.0
Disagree	5.6	5.0
Would need tech support to use DOT Diary		
Agree	1.9	5.0
Neutral	3.7	3.3
Disagree	94.5	91.6
DOT Diary functions are well integrated		
Agree	64.8	71.6
Neutral	29.6	21.7
Disagree	5.6	6.7
DOT Diary has too much inconsistency		
Agree	11.2	6.6
Neutral	13.0	20.0
Disagree	75.9	73.3
Most would learn to use DOT Diary quickly		
Agree	88.9	91.7
Neutral	5.6	5.0
Disagree	5.6	3.3
DOT Diary is cumbersome to use		
Agree	11.1	18.3
Neutral	22.2	18.3
Disagree	66.6	63.3
Felt confident using DOT Diary		
Agree	87.0	81.7
Neutral	13.0	11.7
Disagree	0.0	6.7
Needed to learn a lot to use DOT Diary		
Agree	1.9	5.0
Neutral	11.1	8.3
Disagree	87.0	86.7
Median SUS Summary Score (IQR)	77.5 (70–90)	80 (72.5–87.5)
SUS summary rating		
Best imaginable	29.6	23.3
Excellent	16.7	31.7
Good	42.6	26.7
Okay	11.1	16.7
Poor	0.0	1.7

**Fig. 3:**
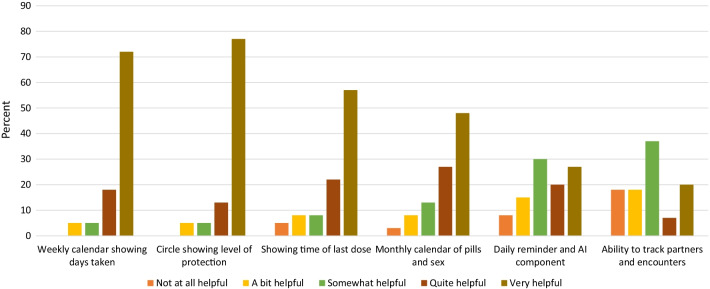
Attitudes towards various components of the DOT Diary app

Participants randomized to the intervention arm were also asked to rate various components of the app; data are presented for the 60 participants attending the week 24 visit (Fig. 3). Overall, the most popular features of the app were the weekly calendar that showed the days the pill was taken, and the infographic that showed the level of protection achieved based on the number of pills taken. Less important to the participants were the daily reminder to take the pill, AI component that recorded pill taking, and the ability to track sex partners and encounters. Overall, 70% of the participants missed taking the pill with the app at least once; 56% said it was because they didn’t have their phone, 48% forgot to use the app, 39% because their phone ran out of battery, 30% because they were in a public place, 30% didn’t have time, 22% because they didn’t want to be seen using the app, and 14% because they were bothered by using the app. When asked whether DOT Diary had an effect on their daily PrEP use, 70% said it made them more likely to take PrEP daily, while 30% said it had no effect on their PrEP use. When asked if the micro-incentive helped with taking daily PrEP, 58.3% said yes, 31.7% said no, and 10% said they didn’t know or were unsure. The control participants were also asked if the micro-incentives for doing a daily activity helped them take daily PrEP; 60.7% said yes, 35.7% said no, and 3.6% were unsure.

### Secondary Objectives: Coverage of Sex Acts and DOT Diary Use Patterns

Among 66 participants in the intervention arm, 42 participants made sexual diary entries on an average of 4.1 (SD 5.4) days. Among 30 men reporting anal sex in their diaries, 93.0% reported taking PrEP using the app for 4–7 days preceding these episodes. Among 34 men reporting sexual episodes other than penetrative anal sex, 95.0% reported taking PrEP using the app for the 4–7 days preceding these episodes. However, there appeared to be substantial under-reporting of sexual practices using the diary compared with what was reported in the CASI. Of the 99 12- and 24-weeks CASI questionnaires where men in the intervention condition reported having had anal sex in the previous 12 weeks, only 35.4% reported anal sex at least once using the app’s sexual diary.

**Fig. 4:**
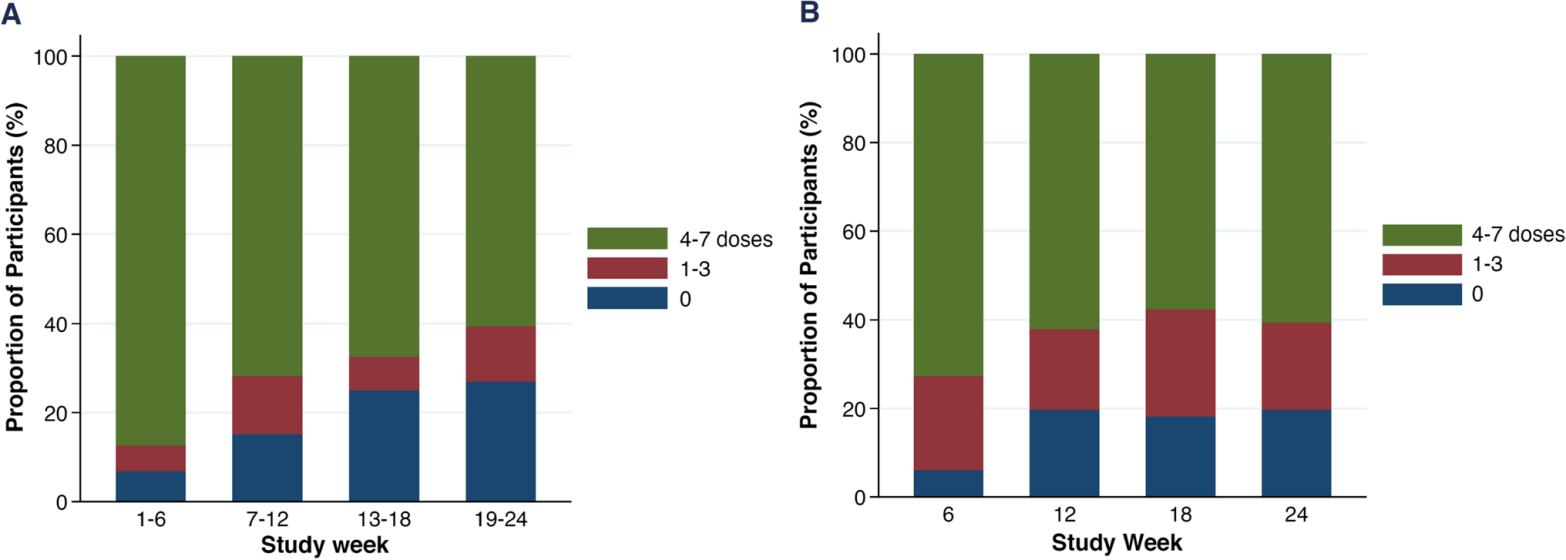
**A** Doses taken per week as reported using the app, by study week. **B** Doses taken per week as measured by TFV-DP levels, by study week

Overall, a median of 77.5% of all expected doses were taken with the DOT Diary app. The frequency of pill taking using the app is shown in Fig. 4A. Of note, there was a significant and monotonic decline in the mean number of pills taken per week using the app by study month, ranging from 5.8 pills in the first 6 weeks to 4.1 pills taken during the last 6 weeks (t = − 7.033, p < 0.001). However, a decline was not seen in TFV-DP levels over time in the participants (Fig. 4B), suggesting increased under-utilization of the app over time for pill taking. Use of the app declined between visits; in a logistic regression analysis for any use of the app between visits, the odds ratio was 0.92 per week (95% CI 0.90–0.95).

## Discussion

The DOT Diary app showed a high level of concordance with FTC-TP and TFV-DP levels (91.0% and 85.3%, respectively), suggesting that this method of measuring adherence is substantially better than self-reported measures [17, 36–38], which often over-report adherence levels because of social desirability [39–42]. Segmental hair levels provide a benefit over DBS levels because they can be used to measure PrEP use over a longer period of time, with drug levels from each centimeter of hair reflecting adherence over previous 4-week periods [43]. However, DOT Diary offers advantages over both measures, because it allows both for longitudinal measurement of adherence over extended periods of time, as well as providing greater granularity on patterns of adherence; measuring hair levels also requires trained personnel and a clinic visit. The DOT Diary tool may be useful in probing patterns of non-adherence with participants or patients, as well as in measuring PrEP adherence with event-based or 2-1-1 dosing [3], given the inclusion of the sexual diary in the DOT Diary app. Overall, 85% of the United States population and 95% of people under 50 years own a smartphone [44], making this a viable method for measuring PrEP adherence in clinical trials or clinical practice.

Overall, the app was well-received by study participants, with more than 90% indicating that they were mostly or very satisfied with it, that they would use the DOT Diary app in the future, and that the app helped them in taking PrEP. Two-thirds of participants reported that having a daily reminder to take the pill with the AI component was at least somewhat helpful in taking PrEP. While 77.5% of expected doses were taken with the app, use of the app appeared to decline somewhat over the 26-week period, suggesting possible fatigue with using the app. This may present an even greater problem if visits are spaced out to be less frequent, as could occur in clinical trials or clinical practice. A similar decline in use of a mobile app to monitor daily PrEP adherence and sexual behavior was seen in the AMPrEP study [45]. Additional features, such as gamification, might improve the persistence of app usage [46], and is being built into a follow-up app that includes the most desired features of DOT Diary, for use for both daily and 2-1-1 PrEP.

Clinic- or home-based DOT has been shown to improve adherence to antiretrovirals for treatment [21, 24], and the AiCure platform of a-DOT has previously shown improved adherence in studies of anticoagulation therapy [47] and treatment for schizophrenia [48], as well as high levels of adherence to hepatitis C treatment [49]. However, in this study, we did not see improved adherence in the intervention arm compared with the control arm. It is possible that providing the micro-incentives to both arms of the study masked any benefit from the DOT Diary app alone, as incentives have been previously shown to improve some health behaviors [50–54], and approximately 60% of both the intervention and control participants stated that getting a micro-incentive improved their daily pill taking. Nearly all participants also stated that participation in the study improved their self-confidence in taking PrEP, so the counseling provided to both study arms may have similarly obscured differences between the two arms. In addition, any PrEP pill-taking was relatively high and consistent in both arms of the study (approximately 90%), and protective levels were associated with condomless anal sex; therefore, it is possible that we were unable to improve on the adherence among participants who may have had relatively lesser risk. Another possibility is that the frequency of visits in both arms (every 6 weeks) helped maintain higher levels of adherence, as more frequent visits were associated with higher levels of adherence among young MSM [55].

Although there was no difference between study arms, drug levels remained fairly consistent over a 6-month period, rather than dropping off, as has been seen in some other studies in young MSM [55, 56]. Data are mixed on whether reminders are effective in improving adherence for medication. Haberer and colleagues tested a short message service (SMS)-based intervention for Kenyan women on PrEP and found it to be ineffective in promoting adherence [57]. On the other hand, several meta-analyses of the impact of SMS text reminders on adherence found that text messages increased adherence to HIV medications [58–60] as have studies in resource-limited settings [61] and among youth living with HIV [62]. More recently, studies have also demonstrated that reminders increase PrEP adherence [63]. In our study, both arms of the study were given a form of reminders, as the control condition also participated in daily health-related app use. These reminders may have contributed to high persistence of any PrEP use throughout the study.

Although protective levels of PrEP were only maintained throughout the study by more than half of the participants in either arm, it is possible that many participants were taking PrEP appropriately to cover sexual acts, so-called “PrEP-effective adherence” [64]. In fact, for those men using the sexual diary, 91.5% of receptive anal sex and 96.6% of insertive anal sex acts were covered with PrEP, according to the DOT Diary app. However, relatively few sexual episodes were reported using the sexual diary, making it difficult to interpret PrEP-effective adherence for all sex acts in the intervention group. Participants may not have reported sexual activity because they did not find it useful and reporting was not required for use of DOT Diary; tracking sexual encounters was of lesser desirability in our survey of participants. In the AMPrEP study, agreement between sexual behavior recorded in the app and reported in quarterly questionnaires was higher [45], possibly due to the app asking a daily question about sexual activity. Lack of full functionality of the sexual diary in DOT Diary that would have provided summary trends of various types of sexual activity, a feature requested in focus groups, may improve sexual diary use. A sexual diary may also be more desirable when needing to track 2-1-1 PrEP use; this study only evaluated daily PrEP. We will be assessing a new iteration of this app with full functionality, that will be used for both daily and 2-1-1 PrEP, and will allow for a comparison of use between the two.

Protective levels of TFV-DP were significantly lower in participants enrolled in Atlanta, and in Black/African American study participants in multivariable analysis. PrEP use is substantially less common in Atlanta than in San Francisco [65], perhaps influencing PrEP use differences between the two cities, as having a PrEP user in one’s social network is associated with being further along the PrEP continuum [66]. However, in the US, PrEP uptake is also significantly less common in Black/African American than white MSM [67] and despite improvements in PrEP uptake among Black/African Americans, disparities remain [68]. Several studies have demonstrated lower PrEP adherence among Black/African American than white participants [55, 69]; the reasons for this are likely multifactorial. A study of DOT-administered PrEP found that DBS levels were 14% lower among Black/African Americans than among white participants [22], suggesting that biological differences in PrEP measurement may also contribute to the PrEP adherence discrepancies. However, other studies point to structural factors and institutional factors, such as structural racism and homophobia in health care settings, lack of access to quality health care and having a trusted primary care provider, lower rates of health insurance, marketing of PrEP to white MSM, and PrEP-related stigma as potential drivers of lower adherence among Black/African American MSM [70–72]. Culturally tailored interventions, such as the integrated comprehensive risk counseling and case management offered in HPTN 073 can overcome some of these obstacles [73], although more is needed to address these structural barriers [74]. These issues must be addressed to maximize PrEP’s impact in reducing HIV acquisition in those who most need PrEP in the US.

### Limitations

This study has several limitations. The study was conducted only among MSM and only at two study sites, which may limit the generalizability of findings to other demographic, geographic, and clinical settings. However, one of the two sites was in the Southern United States, where HIV infections are highest [1] and PrEP uptake lowest [65]. The correlation between TDF/FTC ingestion and drug levels at 6 weeks as measured by DBS is not absolute, as there are individual differences in drug metabolism and red blood cell turnover that could misclassify levels of adherence [22, 75]. Additional research into user interface, app design, or other app functionality may drive improved engagement in future versions of the app. In order to assess the independent effects of DOT Diary, both arms received micro-incentives; these micro-incentives have the advantage of facilitating app use in research design, and provision to both arms ensures that assessment of outcomes is isolated to the intervention, rather than the micro-incentive, but do come with some limitations. In addition to the reminders incorporated into the health apps used by the control condition and the adherence counseling received at every study visit, incentive provision to both arms may have interfered with our ability to measure DOT Diary’s impact.

## Conclusions

Our study demonstrated that DOT Diary could be used to accurately measure PrEP ingestion in a diverse cohort of young MSM. The app was highly acceptable to users, almost all of whom stated they would use the app in the future, were it available. This suggests that such apps could have a role in real-time measurement of adherence for both clinical trials and clinical practice. The app did not improve adherence in this cohort, and this may represent a genuine lack of effect or be related to a number of potential explanations relating to study design. Newer generations of apps that build upon the most desirable features of DOT Diary are being developed and tested for their ability to improve PrEP uptake and adherence.

## Data Availability

De-identified data available upon request.

## Code Availability

Available upon request.
